# *Streptococcus suis* meningitis complicated with communicating hydrocephalus: complete clinical and radiological resolution with non-neurosurgical management—A case report

**DOI:** 10.3389/fmed.2026.1888345

**Published:** 2026-08-28

**Authors:** Mei Qi, Yaqing Zeng, Bofu Ruan, Qin Wang

**Affiliations:** 1 Xiaolan Clinical Institute of Shantou University Medical College, Zhongshan, Guangdong, China; 2 Department of Neurology, Xiaolan People’s Hospital of Zhongshan (The Fifth People’s Hospital of Zhongshan), Zhongshan, Guangdong, China

**Keywords:** case report, communicating hydrocephalus, metagenomic next-generation sequencing, non-neurosurgical management, *streptococcus suis* meningitis

## Abstract

**Background:**

*Streptococcus suis* (*S. suis*) meningitis is a severe zoonotic central nervous system infection, with permanent sensorineural hearing loss as its most common long-term sequela. Hydrocephalus is an extremely rare and underrecognized complication of *S. suis* meningitis, and no case of communicating hydrocephalus secondary to *S. suis* meningitis managed successfully with non-neurosurgical therapy has been formally documented.

**Case presentation:**

A 52-year-old man presented with 8 days of progressive dizziness and gait imbalance. Contrast-enhanced magnetic resonance imaging (MRI) confirmed communicating hydrocephalus with diffuse leptomeningeal enhancement and no ventricular obstruction. Cerebrospinal fluid (CSF) cultures remained persistently negative, while metagenomic next-generation sequencing (mNGS) identified *S. suis* within 48 h with a relative abundance of 92.60%. Guideline-recommended targeted antimicrobial therapy combined with short-course adjunctive dexamethasone achieved complete clinical and radiological resolution without neurosurgical intervention. The patient remained asymptomatic with intact bilateral hearing at the 10-month follow-up.

**Conclusion:**

To our knowledge, this is the first reported case of *S. suis* meningitis-associated communicating hydrocephalus successfully managed without neurosurgery. Accurate hydrocephalus subtyping via contrast-enhanced MRI and rapid pathogen identification via mNGS may help inform clinical decision-making regarding the potential for non-neurosurgical management in carefully selected patients.

## 1 Introduction

*Streptococcus suis* (*S. suis*) is a major zoonotic pathogen posing a substantial public health burden, especially in swine-farming endemic regions of China and Southeast Asia (1, 2). Meningitis is the most common clinical manifestation of invasive *S. suis* infection, accounting for over 50% of reported human cases (3, 4). It is characterized by acute onset, rapid progression, and high long-term morbidity; permanent sensorineural hearing loss affects more than half of survivors (5–7).

Hydrocephalus is a recognized complication of adult community-acquired bacterial meningitis (8), occurring in approximately 4.5% of cases, over 96% of which are the communicating subtype (9). However, this complication remains under-recognized in *S. suis*-specific literature: the largest systematic review of 913 *S. suis* meningitis patients does not list hydrocephalus as a recognized sequela at all (4). A large prospective cohort identified only 2 *S. suis* cases among 26 meningitis patients with hydrocephalus, further confirming its rarity (9). Previously reported *S. suis*-associated hydrocephalus cases were mostly obstructive subtypes requiring surgical CSF diversion (10, 11). No study has formally described communicating hydrocephalus as a complication or provided evidence for successful non-neurosurgical management.

Pathophysiologically, inflammatory communicating hydrocephalus arises from impaired CSF reabsorption at the arachnoid granulations, and is reversible with effective control of underlying infection (12). Conventional culture-based testing has limited diagnostic yield and often delays definitive etiological diagnosis (13). In contrast, mNGS enables rapid pathogen identification in culture-negative central nervous system infections (14, 15), providing an opportunity to consider non-neurosurgical management. This report describes the first case of *S. suis* meningitis-associated communicating hydrocephalus treated successfully with non-neurosurgical management, conducted in accordance with the CARE guidelines (16). It aims to expand the documented clinical spectrum of *S. suis* infection and provide a reference for clinical decision-making in similar rare cases.

## 2 Case presentation

A 52-year-old de-identified Han Chinese man with no significant past medical history presented with 8 days of progressive dizziness and gait instability. He had no history of hypertension, diabetes, cardiovascular disease, or known drug allergies. He was a non-smoker and non-drinker, with no significant family history of neurological or infectious diseases. He was a local resident with no classical occupational or dietary swine exposure, but had long resided in a swine-farming endemic region of southern China. His symptoms deteriorated progressively despite empiric symptomatic treatment (details unavailable) at a local clinic, and he developed mild headache and nausea on the day of presentation.

### 2.1 Initial assessment

**Figure 1:**
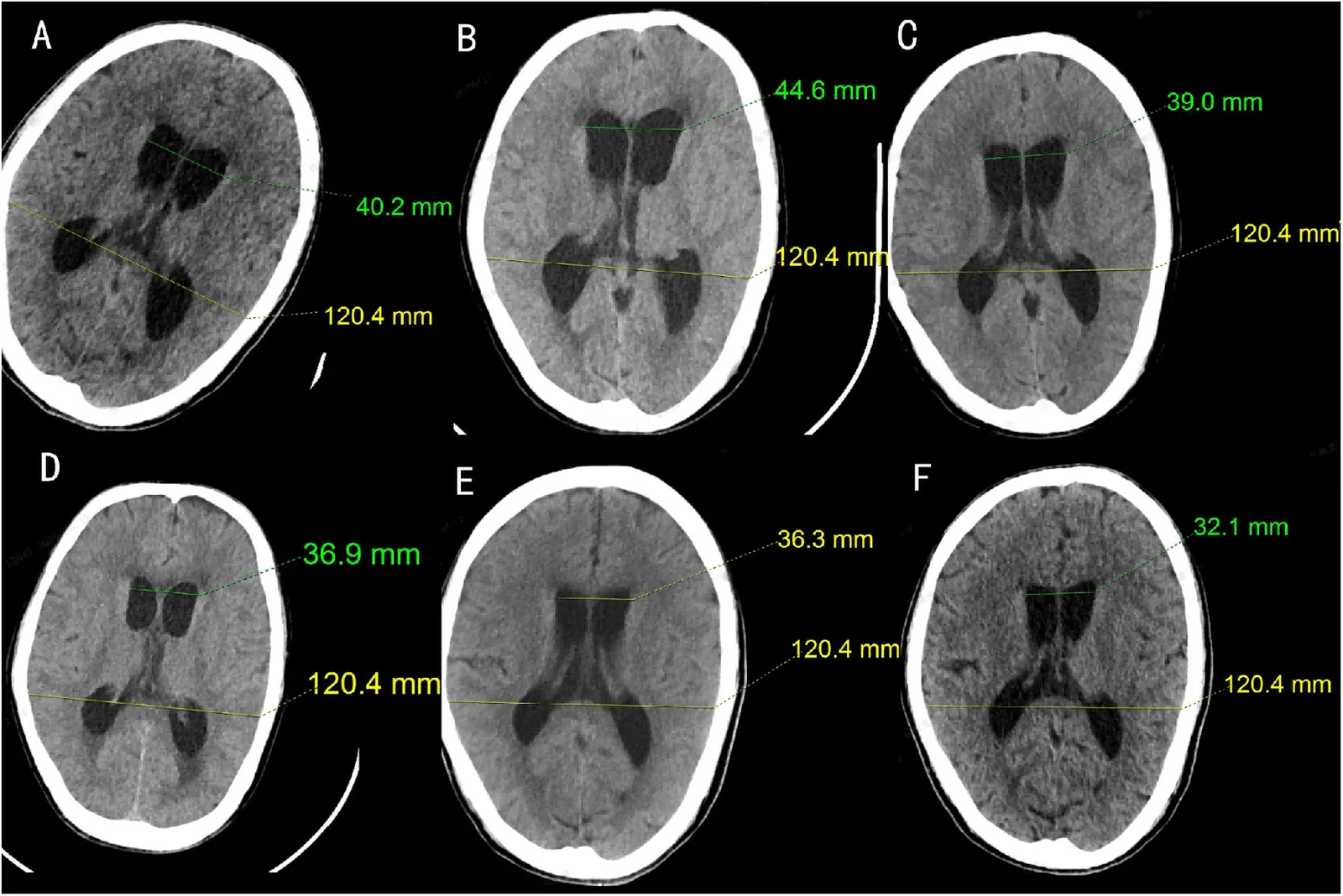
Serial non-contrast cranial CT scans demonstrating the dynamic evolution of hydrocephalus. **(A)** Hospital day 1: marked supratentorial ventricular dilatation with periventricular hypoattenuation (Evans index = 0.334). **(B)** Hospital day 12: transient worsening of ventricular dilatation (Evans index = 0.370). **(C)** Hospital day 19: partial improvement in ventricular size (Evans index = 0.324). **(D)** Hospital day 26: significant reduction in ventricular dimensions with resolution of periventricular hypoattenuation (Evans index = 0.306). **(E)** 3-month follow-up: near-complete resolution of ventricular dilatation (Evans index = 0.301). **(F)** 10-month follow-up: complete normalization of ventricular size with no recurrence (Evans index = 0.267). Evans index = maximum width of the frontal horns/maximum inner diameter of the calvaria (normal < 0.30).

On admission, the patient was afebrile with stable vital signs and a Glasgow Coma Scale (GCS) score of 15, indicating intact consciousness. Neurological examination revealed nuchal rigidity and a positive Romberg sign, with no focal neurological deficits, cranial nerve palsy, or hearing impairment on bedside testing. Initial blood work showed leukocytosis with neutrophil predominance and elevated inflammatory markers; renal function, liver function, and serum glucose were within normal limits. Non-contrast cranial computed tomography (CT) was performed on admission as initial emergency screening. It ruled out acute intracranial hemorrhage and space-occupying lesions and provided preliminary assessment of hydrocephalus severity. It demonstrated marked supratentorial ventricular dilatation with periventricular hypoattenuation (Evans index = 0.334) (Figure 1A), consistent with hydrocephalus.

### 2.2 Diagnostic workup

On hospital day 2, the patient developed transient fever (38.0 °C). Two sets of blood cultures were obtained, and empiric intravenous ceftriaxone (2.0 g every 12 h) was initiated in line with international guidelines for suspected bacterial meningitis (17).

**Figure 2:**
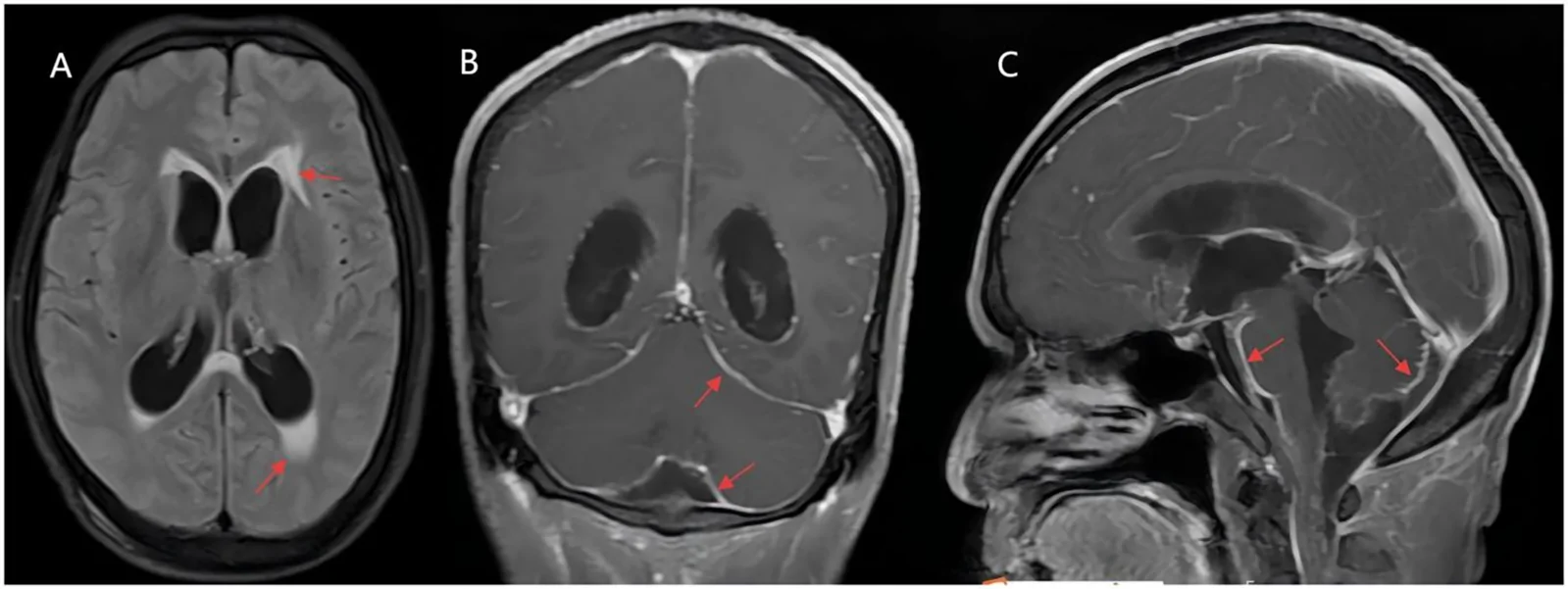
Cranial MRI findings on hospital days 4 and 5. **(A)** Axial T2-FLAIR image (day 4): ventricular dilatation and periventricular hyperintensities (red arrows) consistent with transependymal interstitial edema. **(B)** Coronal and **(C)** sagittal post-contrast T1-weighted images (day 5): diffuse leptomeningeal enhancement (red arrows) involving the basal cisterns, tentorium cerebelli, and brainstem surfaces.

Cranial MRI performed on hospital day 4 confirmed communicating hydrocephalus, with ventricular dilatation and periventricular T2-FLAIR hyperintensities indicative of transependymal interstitial edema. No obstructive lesions were identified in the cerebral aqueduct or fourth ventricle. Post-contrast T1-weighted images on hospital day 5 revealed diffuse leptomeningeal enhancement involving the basal cisterns, tentorium cerebelli, and brainstem surfaces, consistent with active leptomeningitis (Figure 2).

Diagnostic lumbar puncture (LP) was performed on hospital day 6 to identify the pathogen and establish baseline inflammatory status. Given the presence of hydrocephalus, the procedure was performed after multidisciplinary neurological and neurosurgical consultation confirmed its safety. CSF analysis showed typical suppurative changes: elevated opening pressure (240 mmH₂O), pleocytosis (468 × 10⁶/L), markedly elevated protein (6,296.60 mg/L), hypoglycorrhachia (glucose 1.18 mmol/L), and reduced chloride concentration (104.0 mmol/L). Gram staining and both CSF and blood cultures remained negative after 5 days of incubation. Residual CSF was submitted for mNGS, which identified *S. suis* within 48 h with 8,062 uniquely mapped reads and a relative abundance of 92.60%. The high relative abundance of a single pathogen, combined with the typical suppurative CSF profile and clinical presentation consistent with bacterial meningitis, strongly supports a diagnosis of true infection rather than contamination. Contamination was considered unlikely for three reasons. First, a single organism predominated with high relative abundance (>90%), whereas contaminants typically present as mixed flora with low relative abundance. Second, the mNGS result was concordant with the clinical and laboratory picture of acute bacterial meningitis, including marked CSF pleocytosis, profoundly elevated protein, and hypoglycorrhachia. Third, the patient showed rapid clinical and radiological response to pathogen-directed antimicrobial therapy targeting *S. suis.*

The differential diagnosis for this presentation included: (1) bacterial meningitis from other common pathogens (Streptococcus pneumoniae, Neisseria meningitidis, Haemophilus influenzae); (2) tuberculous meningitis, given the subacute 8-day course and communicating hydrocephalus; (3) fungal meningitis, particularly Cryptococcus neoformans; (4) carcinomatous meningitis; and (5) non-infectious causes of communicating hydrocephalus, such as idiopathic normal pressure hydrocephalus or post-hemorrhagic hydrocephalus. The acute onset of symptoms, marked CSF pleocytosis with neutrophil predominance (468 × 10⁶/L, 73.5% neutrophils), profoundly elevated protein (6,296.60 mg/L), and hypoglycorrhachia (1.18 mmol/L) strongly favored acute bacterial meningitis over other etiologies. The subsequent identification of *S. suis* by mNGS with high relative abundance established the diagnosis.

### 2.3 Treatment and monitoring

On hospital day 8, a definitive diagnosis of *S. suis* meningitis complicated by communicating hydrocephalus was established. After multidisciplinary consultation, a non-neurosurgical management strategy was elected, given the patient's preserved consciousness, confirmed communicating subtype, and absence of herniation signs.

The antimicrobial regimen was optimized to combination therapy with ceftriaxone (2.0 g every 12 h) and linezolid (600 mg every 12 h). The decision to add linezolid was an individualized empiric choice based on the following considerations: First, given the severity of the presentation (communicating hydrocephalus with active leptomeningitis) and the absence of culture and susceptibility data, combination therapy with two agents having robust CNS penetration was chosen to maximize bactericidal coverage. Ceftriaxone remains the first-line agent for S. suis meningitis. However, emerging reports of reduced *β*-lactam susceptibility among *S. suis* isolates from swine and human sources in endemic regions of China raised concern for empiric monotherapy failure in this severe, complicated case. Second, linezolid achieves consistently high CSF concentrations throughout the dosing interval, providing reliable bactericidal activity in the CSF compartment—a particularly important consideration in this patient with impaired CSF reabsorption due to communicating hydrocephalus. Third, the non-ototoxic safety profile of linezolid was considered a key advantage in this case. Permanent sensorineural hearing loss is the most common long-term sequela of *S. suis* meningitis, affecting more than half of survivors (6, 18, 19). The addition of a non-ototoxic agent to the regimen was intended to mitigate this high risk of a devastating permanent neurological complication. Of note, linezolid is not considered standard first-line therapy for *S. suis* meningitis, and this decision was made on an individualized empiric basis given the specific circumstances of this case. Short-course adjunctive dexamethasone (0.15 mg/kg every 6 h) was administered for 4 days to reduce leptomeningeal inflammation and mitigate arachnoid adhesion risk, consistent with guideline principles for acute bacterial meningitis (17, 20).

Inflammatory communicating hydrocephalus is inherently reversible with effective infection control, but carries a risk of conversion to obstructive hydrocephalus during the active inflammatory phase. Accordingly, four diagnostic lumbar punctures were performed at distinct clinical time points, primarily for monitoring purposes rather than therapeutic CSF drainage, each with explicit clinical triggers:

First LP (hospital day 6): Diagnostic procedure to identify the pathogen and establish baseline inflammatory status, performed after multidisciplinary neurological and neurosurgical consultation to confirm procedural safety. The opening pressure was elevated at 240 mmH₂O, consistent with active leptomeningitis and impaired CSF reabsorption.

Second LP (hospital day 12): Performed in response to transient ventricular enlargement detected on CT, to differentiate uncontrolled infection from the peak inflammatory response. Cranial CT was obtained first to exclude obstructive conversion and verify safety. The opening pressure had decreased to 160 mmH₂O, indicating improved CSF circulation despite transient ventricular dilatation on imaging.

Third LP (hospital day 19): Performed to quantify inflammatory resolution and guide safe antibiotic discontinuation in this rare presentation, minimizing the risk of relapse from insufficient treatment duration. Concurrent CT served as pre-procedural safety assessment and radiological follow-up. The opening pressure remained stable at 160 mmH₂O.

Fourth LP (hospital day 25): Pre-discharge baseline assessment for long-term follow-up comparison, with an opening pressure of 150 mmH₂O within the normal range.

Serial LPs served primarily as an individualized diagnostic monitoring tool; any adjunctive benefit from inflammatory CSF drainage cannot be definitively quantified in this single case. Dynamic changes in all CSF and blood parameters are summarized in Table 1.

**Table 1:** Dynamic laboratory parameters.

Category	Parameter	Day 1	Day3	Day 6	Day 12	Day 19	Day 25	Reference range
Blood routine examination	WBC(10⁹/L)	19.17	10.20	8.51	7.35	8.59	9.12	3.5–9.5
	NEUT(%)	91.2	81.3	73.5	64.8	59.1	60.8	40–75
	PLT(10⁹/L)	449	419	455	384	211	227	125–350
	Hb(g/L)	113	111	116	114	108	117	130–175 (male)
	HCT(%)	0.356	0.358	0.356	0.364	0.331	0.360	40–50 (male)
	RBC(10¹²/L)	5.93	5.86	6.04	5.88	5.12	5.55	4.3–5.8
CSF analysis	Appearance			Turbid	Transparent	Transparent	Transparent	Transparent
	Opening pressure (mmH₂O)			240	160	160	150	80–180
	WBC count (×10⁶/L)			468	259	79	30	0–8
	Protein content (mg/L)			6,296.60	1,538.50	859.94	455.0	150–450
	Glucose (mmol/L)			1.18	2.70	2.76	2.80	2.5–4.4
	Chloride (mmol/L)			104.0	117.0	120.0	122.0	120–130
	Culture			Negative	Negative	Negative	Negative	
mNGS				*S.suis*				

WBC, white blood cell; NEUT, neutrophil granulocyte; PLT, platelet; Hb, hemoglobin; HCT, hematocrit; RBC, red blood cell; CSF, cerebrospinal fluid; *S.suis*, *Streptococcus suis*; mNGS, metagenomic next-generation sequencing.

### 2.4 Outcome and follow-up

**Figure 3:**
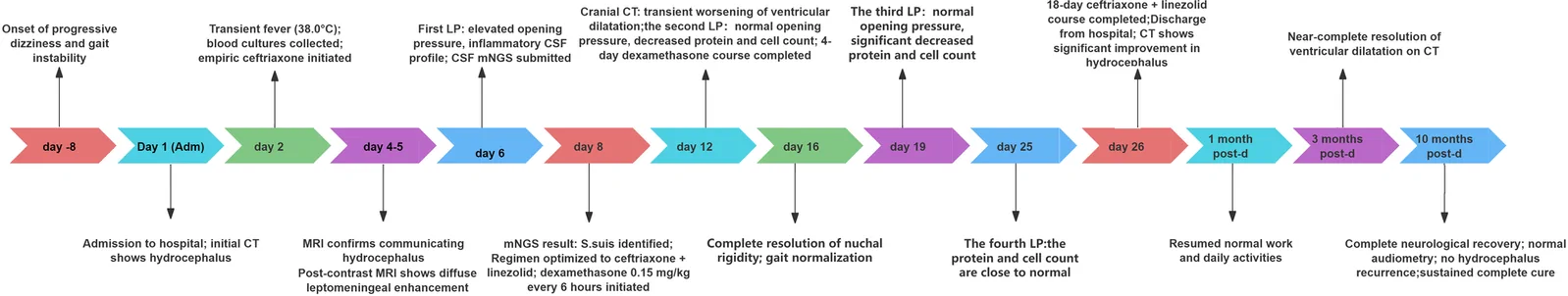
Timeline of clinical manifestations, diagnostic workup, and therapeutic interventions across the full episode of care.

The overall timeline of diagnosis, treatment and clinical evolution is summarized in Figure 3.The patient achieved substantial clinical improvement by hospital day 16, with complete resolution of nuchal rigidity and full normalization of gait and balance. Pre-discharge cranial CT was obtained on hospital day 26 to establish the radiological baseline for long-term follow-up. It showed reduced ventricular size (Evans index = 0.306) and complete resolution of periventricular edema. The total duration of antimicrobial therapy was 25 days, with 18 days of linezolid combination therapy. No clinically significant hematological toxicity attributable to linezolid was observed. Complete serial hematological monitoring, including platelet counts, hemoglobin, hematocrit, and erythrocyte parameters, was performed throughout treatment. Platelet counts and white blood cell counts remained within normal limits. Mild anemia was present at baseline, consistent with infection-associated anemia of chronic disease, and remained stable during treatment with evidence of improvement by the end of therapy (Table 1).

At the 3-month follow-up, cranial CT showed near-complete resolution of ventricular dilatation (Evans index = 0.301). At the 10-month follow-up, the patient remained entirely asymptomatic with no hydrocephalus recurrence. Formal audiometry confirmed normal bilateral hearing, and cranial CT showed complete normalization of ventricular dimensions (Evans index = 0.267). Serial CT findings of hydrocephalus progression are shown in Figure 1.

## 3 Discussion

To the best of our knowledge, this is the first formally documented case of communicating hydrocephalus secondary to *S. suis* meningitis treated successfully with non-neurosurgical management.

### 3.1 Clinical novelty

Hydrocephalus is a rare complication of adult bacterial meningitis, and its occurrence in *S. suis* meningitis remains markedly underrecognized. The largest systematic review of 913 *S. suis* meningitis patients does not list hydrocephalus as a recognized sequela (4), and a large prospective cohort identified only 2 *S. suis* cases among 26 meningitis patients with hydrocephalus (9). Previously reported *S. suis*-associated hydrocephalus cases were predominantly obstructive subtypes requiring surgical CSF diversion (10, 11), with no published evidence for successful conservative management of the communicating subtype.

Against this background, our case illustrates two important points. First, it confirms that communicating hydrocephalus can occur as a complication of *S. suis* meningitis and that complete clinical and radiological resolution is possible without neurosurgical intervention in carefully selected patients. Second, it illustrates a potential diagnosis-based approach. In this patient, accurate hydrocephalus subtyping via contrast-enhanced MRI, combined with rapid pathogen identification via mNGS, informed the decision to attempt medical management rather than immediate neurosurgical intervention.

### 3.2 Rationale for non-neurosurgical management

The feasibility of non-neurosurgical observation is based on the reversible nature of inflammatory communicating hydrocephalus. Obstructive hydrocephalus is caused by physical ventricular blockage. In contrast, meningitis-associated communicating hydrocephalus arises from impaired CSF reabsorption at the arachnoid granulations due to diffuse leptomeningeal inflammation and typically resolves once the underlying infection is controlled (12). In this case, contrast-enhanced MRI confirmed diffuse leptomeningeal enhancement without obstructive lesions, confirming the communicating subtype and supporting an initial trial of medical management.

Rapid definitive diagnosis was critical to this approach. For this culture-negative case, mNGS identified *S. suis* within 48 h, enabling early optimization to pathogen-directed therapy with excellent central nervous system penetration (13–15). A confirmed etiology eliminated the need for broad empiric coverage and obviated the need for empiric surgical intervention for unexplained hydrocephalus.

### 3.3 Applicability of this strategy

The favorable outcome in this case was attributed to four key factors: preserved consciousness throughout the clinical course, isolated communicating hydrocephalus without obstructive features, early targeted antimicrobial therapy initiated before irreversible arachnoid adhesion developed, and absence of significant comorbidities.

Non-neurosurgical management is only appropriate for carefully selected patients. Surgical CSF diversion remains the first-line treatment for obstructive hydrocephalus, neurological deterioration, or signs of impending brain herniation (12). Based on the single case, we tentatively suggest the following considerations for potential non-neurosurgical management, which require validation in larger case series. Non-neurosurgical management may be cautiously considered only when all of the following features are present:

− Contrast-enhanced MRI confirms communicating hydrocephalus without ventricular obstruction;
− Stable clinical status with no signs of brain herniation;
− Rapid definitive microbiological diagnosis enabling early targeted antimicrobial therapy;
− Availability of urgent neurosurgical backup if clinical deterioration occurs.

In line with the ESCMID guideline, routine repeated lumbar punctures (LPs) and intracranial pressure monitoring are not recommended for uncomplicated bacterial meningitis, as solid evidence is lacking and potential harm may occur (17). However, this case was complicated by secondary communicating hydrocephalus, which represents a severe, complicated presentation outside the scope of uncomplicated meningitis. In this specific context, serial diagnostic LPs were adopted as an individualized monitoring strategy to track inflammatory resolution and inform the duration of antimicrobial treatment (21).

For clarity, the term “non-neurosurgical management” in this report specifically refers to treatment without neurosurgical CSF diversion procedures (e.g., ventriculoperitoneal shunt, external ventricular drain). Diagnostic lumbar puncture is a routine diagnostic procedure in neurology practice and is not categorized as a neurosurgical intervention. The serial LPs implemented in this case were tailored to this rare complicated presentation and should not be generalized to standard clinical practice.

### 3.4 Study limitations

This single-case report has several inherent limitations.

First, all CSF and blood cultures remained negative, which precluded *in vitro* antimicrobial susceptibility testing, *S. suis* serotyping, and multilocus sequence typing, limiting the ability to correlate clinical phenotype with strain virulence factors. Additionally, species-specific PCR was not available at our institution for confirmatory testing, which represents a diagnostic limitation of our setting. Nevertheless, pathogen diagnosis was supported by mNGS with high relative abundance and strong clinico-laboratory concordance, and the selected antimicrobial regimen aligns with the documented susceptibility profile of *S. suis* isolates in endemic regions of China (18).

Second, follow-up contrast-enhanced cranial MRI was not obtained, which would have provided definitive confirmation of complete leptomeningeal enhancement resolution. Serial cranial CT confirmed progressive normalization of ventricular dimensions, and the patient remained asymptomatic at long-term follow-up.

Third, serum and CSF linezolid concentrations were not monitored, limiting the ability to provide optimized dose recommendations for this specific clinical scenario.

Fourth, only the Evans index was reported for radiological quantification of ventriculomegaly; the bicaudate index was not calculated as the selected CT images were not acquired at the standard anatomical plane for this measurement, which limits the comprehensiveness of radiological evaluation.

Fifth, serial LPs served as an individualized diagnostic and monitoring strategy for this complicated case rather than a standardized recommended regimen. The necessity of the third and fourth LPs can be challenged, and their independent clinical benefit cannot be quantified in a single case report. Additionally, although all cranial CT examinations were performed for explicit clinical indications (including pre-LP safety assessment and evaluation of clinical fluctuation), repeated radiation exposure remains an inherent limitation of this case.

Sixth, the independent impact of dexamethasone on clinical and radiological outcomes cannot be definitively determined, and its administration timing was not fully aligned with guideline recommendations for empiric use.

Finally, the favorable outcome is primarily attributable to effective control of the underlying infection, as inflammatory communicating hydrocephalus is inherently reversible once inflammation is suppressed. The independent contribution of the monitoring strategies used here cannot be confirmed, and there is a risk of overinterpreting the efficacy of the management approach. Further prospective multicenter case series are needed to validate these findings and define standardized patient selection criteria.

## 4 Conclusion

This case expands the documented clinical spectrum of invasive *S. suis* infection by describing the first reported case of communicating hydrocephalus secondary to *S. suis* meningitis managed successfully without neurosurgical intervention. In this carefully selected patient with confirmed communicating subtype, preserved consciousness, and rapid definitive pathogen diagnosis, complete clinical and radiological resolution was achieved with guideline-concordant antimicrobial and adjunctive therapy.

For clinical practice in endemic regions, *S. suis* should be routinely included in the differential diagnosis of adult bacterial meningitis even in the absence of classical swine exposure. Contrast-enhanced cranial MRI is critical for hydrocephalus subtyping to guide subsequent management decisions. The non-neurosurgical strategy described here represents an individualized approach for rare complicated cases, rather than a generalized treatment recommendation. Larger prospective studies are required to validate the safety and efficacy of this approach and to establish standardized patient selection criteria.

## 5 Patient perspective

The patient reported significant improvement in dizziness and gait instability after 8 days of targeted combination therapy. He stated that he had been anxious about the prospect of brain surgery on admission, and felt fortunate to achieve full recovery without neurosurgical intervention. At discharge, he reported complete resolution of symptoms and full independence in daily activities. At the 10-month follow-up, he confirmed that he had returned to normal work and daily life with no residual symptoms or hearing impairment.

## Data Availability

The datasets presented in this study can be found in online repositories. The names of the repository/repositories and accession number(s) can be found in the article/Supplementary Material.
